# Effectiveness of Adult Chest Compressions during Resuscitation Performed by Children Aged 10–14 Years under Simulated Conditions

**DOI:** 10.3390/jcm13195933

**Published:** 2024-10-05

**Authors:** Piotr Konrad Leszczyński, Wiktoria Ciołek, Justyna Cudna, Tomasz Ilczak

**Affiliations:** 1 Department of Medical and Health Sciences, University of Siedlce, 08-110 Siedlce, Poland; 2 Department of Emergency Medicine, Faculty of Health Sciences, University of Bielsko-Biala, Willowa 2, 43-309 Bielsko-Biała, Poland; 3 European Pre-Hospital Research Network, Nottingham NG11 8NS, UK

**Keywords:** teaching, education, effectiveness, resuscitation, basic life support, children, chest compression

## Abstract

**Introduction:** Numerous educational programs recommend implementing the teaching principles of BLS from an early age. The aim of this study was to evaluate selected parameters of the quality of resuscitation performed by children aged 10–14 years during simulated circulatory arrest in an adult. **Materials and Methods:** The project involved four stages, culminating in students performing thoracic compressions on an adult simulator for 2 min. A digital analysis of the quality, depth, relaxation and rate of compressions allowed us to formulate results and conclusions. The authors’ proprietary questionnaire form allowed for the correlation of criteria such as age, gender, body mass and past experience in first aid training of the participant. **Results:** A total of 149 girls and 130 boys were studied. The mean age was 12 years (SD ± 1.41). A directly proportional increase in body mass with participant age was observed (*p* < 0.000). Children as young as 10 years old achieved only 24.13% quality, while those at the age of 14 demonstrated a more than doubled value (67.61%). The minimum depth of chest compressions recommended for an adult (5–6 cm) was not reached in any age group. Girls from all grades achieved a mean quality of 44.69% (SD ± 32.57), while boys achieved a score of 60.23% (SD ± 31.74). On the other hand, in the case of evaluating thoracic relaxation, a significantly better result was achieved by girls compared to boys (66.14% vs. 56.78%; rho-Spearman test for *p* = 0.011). **Conclusions:** Age, sex and body mass play important roles in the quality of resuscitation provided by children. None of the age groups studied achieved the minimum mean depth during adult thoracic compressions under simulated conditions. It is recommended to modify school-based BLS classes to better match the exercises to students’ predispositions.

## 1. Introduction

Sudden out-of-hospital cardiac arrest (OHCA) is one of the most common causes of death in Europe [1]. The morbidity of cardiovascular etiology constitutes a significant problem for health care systems around the world. Despite professional emergency services, mortality due to cardiac arrhythmias, stroke and acute coronary syndrome is high [2,3,4,5]. Recent studies indicate that the chances of victim survival can be increased to as much as 50–70% if witnesses to an incident correctly recognize OHCA and immediately implement a management regimen called basic life support (BLS) [6]. Activities such as assessing consciousness and breathing, calling an ambulance, performing chest compressions and breaths and using an Automated External Defibrillator (AED) should be familiar to every member of society. For years now, global scientific societies have been pointing out the effectiveness of the simple activities included in the “survival chain” [7]. Early cardiopulmonary resuscitation (CPR) should be performed by everyone, even children.

In recent years, the important role of good-quality chest compressions has been emphasized, driven by the need to maintain perfusion in key internal organs [8]. Correctly performed chest compressions during CPR can generate carotid artery pressures of 30–35 mmHg. For optimal results, several conditions must be met, as stated in the European Resuscitation Council guidelines. The patient should be placed on their back on a hard surface with their chest exposed. The rescuer should place laced hands at the lower part of the sternum so that one base of the hand touches the body of the victim and the other is on the dorsal part of the dominant hand. Compressions should be performed to a depth of 5–6 cm (in adults), allowing the chest to return to its physiological shape (relaxation) after each compression, without pulling the hand away from the sternum. Minimizing pauses in compressions and performing them at a rate of 100–120 per min results in the most effective blood perfusion. Current scientific research highlights the direct correlation between good-quality resuscitation and patient survival.

Education in cardiopulmonary resuscitation and rescue procedures is provided both in traditional forms and with the use of modern teaching techniques, such as e-learning and virtual reality [9,10,11,12]. Remote learning solutions are an alternative when training is actually limited (e.g., by a pandemic) or involves a large number of people [13,14]. First aid knowledge and skills fade after just a few months, so the preferred principle is “lifelong learning”. In Poland, a compulsory subject called “Education for Security” (Polish: Edukacja dla bezpieczeństwa) has been introduced in the school education system, implemented at the elementary and secondary school levels [15]. Classes are usually taught by teachers, sometimes assisted by emergency medical service professionals [16]. However, due to the high cost of training equipment and the lack of a requirement for paramedics to attend such classes, school-based lessons may not be sufficient for students to acquire proper BLS skills.

The basic activity usually taught during educational programs and first aid courses is CPR [17,18]. Correct position and strength of the rescuer are required for optimal-quality chest compressions in an adult. Lack of adequate depth, rate or relaxation during chest compressions result in a significant decrease in their effectiveness and, consequently, a decrease in the victim’s survival rate [19]. Having considered the foregoing, BLS instructors make an ongoing assessment of the student’s performance, most often using a computer analysis of CPR quality on a specialized doll-based simulator [20].

There are few reports in the literature on the quality of resuscitation performed by school-aged children. The European Resuscitation Council’s “Kids Save Lives” program encourages cyclic training for children and adolescents, but no optimal solution has yet been developed to cover the disproportion of the child compared to the adult in need who they must help [21]. This raises the question of whether teaching children to physically perform CPR on an adult can translate into effective assistance in real-world settings. OHCA most often occurs in adults, which is why most first aid classes involve adult resuscitation exercises. This confirms the essence of the question posed.

The authors defined the purpose of this study as evaluating the quality of chest compressions in adults as performed by school-aged (10–15) children under simulated conditions. An attempt was made to identify factors (e.g., age, sex and body mass) that allow children to achieve optimal CPR quality, which may help to better design first aid classes in schools.

## 2. Materials and Methods

This study was conducted on a group of 279 children aged 10–15 from four elementary schools in central Poland. The project was implemented in the 2023/2024 period and received a positive opinion from the Research Ethics Committee No. 2/2024. This study was conducted in four stages.

### 2.1. Stage 1

The authors obtained approval from directors of educational institutions to conduct this study and also submitted an application to the ethics committee. Parental consent was also obtained for the children’s participation in this study, which constituted the primary inclusion criterion. The authors did not receive any refusals from the children’s guardians. The information describes the formula for the course of the test, which involves subjecting a child to several minutes of thoracic compressions on a full-scale adult medical simulator. It was also reported that children would be asked about their age, sex, and body mass, which was necessary for the continuation of the classes. By doing so, children needed to come to class prepared to give honest answers after talking to their parents/guardians. Each meeting with the children was supervised directly by a school teacher.

### 2.2. Stage 2

This stage involved conducting a survey questionnaire among children included in this study. The form addressed issues such as the class the respondent attended; the respondent’s age; the respondent’s sex; the respondent’s body mass [kg]; participation in first aid classes in school; and whether they previously completed a chest compression exercise during school-based first aid classes. At this stage, each participant was assigned an individual number to remain anonymous throughout the study.

### 2.3. Stage 3

Each group participating in this study was given brief instructions on how to perform technically correct chest compressions in an adult. The investigator demonstrated the correct position, depth, rate, relaxation and location of compressions on the medical simulator. For this purpose, the Resusci Ann model from Laerdal (Stavanger Norway) was used with the SimPad control module, providing an evaluated tool for assessing resuscitation [22]. Participants were allowed to ask questions at this stage, but training on a medical simulator was not allowed. This eliminated the confounding factor of better preparation for the practical task as well as the possibility of prior fatigue in the child.

### 2.4. Stage 4

One by one, the children performed continuous chest compressions on a phantom for 2 min without ventilating the victim. In the first minute, they received instructions from the instructor to obtain the best possible CPR result. During the second minute of compressions, the instructor gave no feedback, indicating only the time to complete the task. At each stage of the practical task, participants were allowed to abandon their activities. The following variables were assessed: overall quality of resuscitation [%], rate of compressions per minute, depth of compressions and chest relaxation, mean chest compression rate presented per minute and mean compression depth [mm].

### 2.5. Statistical Analysis

Statistical analysis was performed using SPSS software 29.0 under an OpenSource license, with the significance level set at *p* < 0.05. The following statistical tests were used: The Pearson Chi^2^ test was used. To assess the normality of the distribution in the sociodemographic criteria of the participants, the Shapiro–Wilk test was used. In the absence of normality of the distribution of variables, the rho-Spearman non-parametric test was used.

## 3. Results

### 3.1. The Characteristics of the Study Group

The group consisted of 279 students from four educational institutions. A total of 149 girls and 130 boys from elementary school grades 4–8 were studied. The mean age was 12 years (SD ± 1.41). The mean body mass of the students was 50.71 kg (SD ± 15.28). The majority (*n* = 167; 59.86%) reported that they had already taken part in first aid classes at school in the past. A slightly smaller number of students (*n* = 151; 54.12%) said they had the opportunity to practice chest compressions on a medical model during the class.

### 3.2. Intergroup Differences

The students were divided by age category into subgroups: 10 years (*n* = 53), 11 years (*n* = 62), 12 years (*n* = 51), 13 years (*n* = 63), 14 years (*n* = 46) and 15 years (*n* = 4). Due to the significant difference in the size of the 15-year-old group, it was removed from further analysis, thus ruling out a possible confounding factor from the results. The girl-to-boy ratio was verified, obtaining statistically significant differences according to the Chi square test only in the group of ten-year-olds. The mean body masses were calculated, as well as the first aid classes and chest compression exercises completed so far, as shown in Table 1.

A directly proportional increase in body mass with participant age was observed. The normality of the distribution in the body mass category was not demonstrated (Shapiro–Wilk test; *p* < 0.000); therefore, the rho-Spearman non-parametric test was used to assess the correlation of age to body mass, obtaining *p* < 0.000. The students’ declared prior completion of first aid classes and exercises did not show a similar relationship.

### 3.3. Quality of Chest Compressions

**Figure 1:**
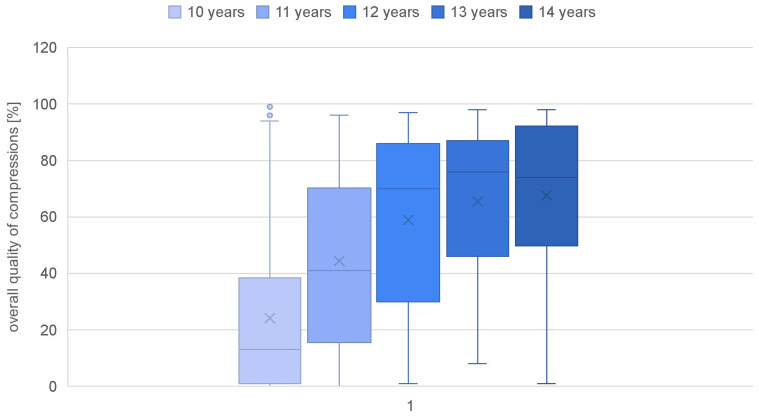
Overall quality of chest compressions in individual age groups.

**Figure 2:**
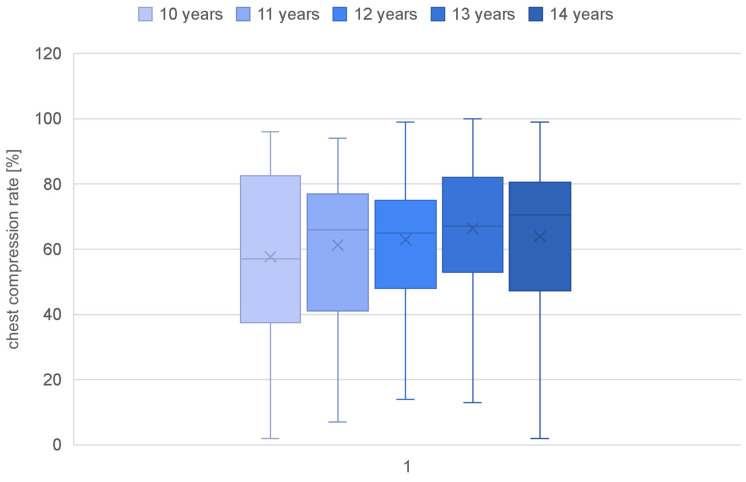
Chest compression rates in specific age groups.

**Figure 3:**
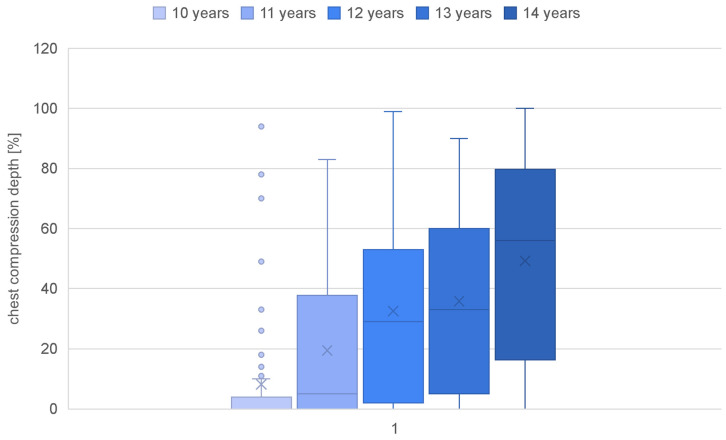
Chest compression depths in individual age groups.

**Figure 4:**
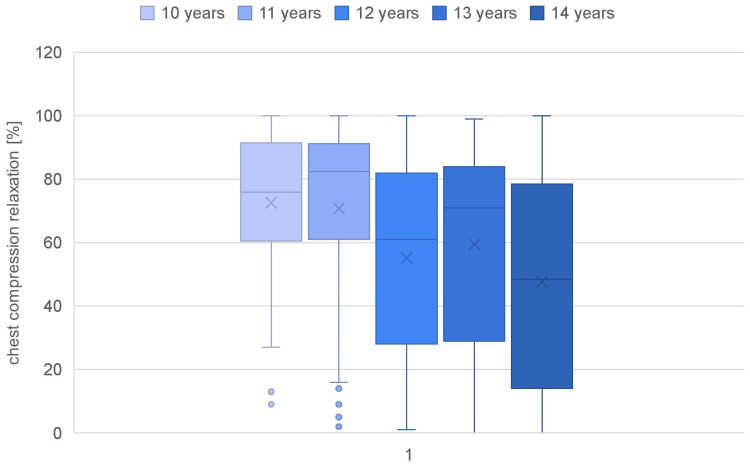
Chest compression relaxation in different age groups.

Parameters such as the overall quality of chest compressions, the rate of compressions (percentage and quantity), the depth of compressions (percentage and quantity), and the quality of relaxation were calculated for each age category (Table 2). Children as young as 10 years old achieved a quality score of only 24.13%, while those at the age of 14 demonstrated a more than doubled value (67.61%) (Figure 1). A statistically significant correlation of age with the overall quality of chest compressions was confirmed (rho-Spearman test; *p* < 0.000). The compression rate in each group was achieved at a similar level, and there was no correlation of this parameter with the age of the study participants (rho-Spearman test; *p* = 0.080) (Figure 2). The mean compression depth of 50–60 mm was not reached in any group. Nevertheless, a significant correlation of this parameter with the student age was observed (rho-Spearman test; *p* < 0.000), confirming that the depth of chest compressions improved with an increasing age (Figure 3). Chest relaxation during CPR was also found to be significantly yet inversely related to the subjects’ age (rho-Spearman test; *p* < 0.000) (Figure 4). The older the student, the worse the chest relaxation was (Table 2).

A further analysis of the acquired data showed that sex determined the overall quality of resuscitation (rho-Spearman test; *p* < 0.000). Girls from all grades achieved a mean quality score of 44.69% (SD ± 32.57), while the boys achieved a score of 60.23% (SD ± 31.74). The rate of chest compressions was independent of sex (rho-Spearman test; *p* = 0.378). However, the depth of compressions was significantly better when performed by boys than by girls (39.10% vs. 19.17%; rho-Spearman test for *p* = 0.000). On the other hand, in the case of evaluating thoracic relaxation, a significantly better result was achieved by girls compared to boys (66.14% vs. 56.78%; rho-Spearman test for *p* = 0.011). The students’ declared past completion of first aid classes correlated significantly with the overall quality of compressions achieved (rho-Spearman test for *p* = 0.031), but this had no effect on the compression rate (rho-Spearman test for *p* = 0.464) and relaxation (rho-Spearman test for *p* = 0.428). In contrast, it differentiated the results in terms of the compression depth (rho-Spearman test for *p* = 0.040). This translates into past CPR exercises performed by students, as those claiming to have experience in this area achieved a higher overall quality of compressions (rho-Spearman test for *p* < 0.000). Still, the thoracic compressions performed did not translate into a better compression rate (rho-Spearman test for *p* < 0.591). A significant correlation was shown for compression depth (rho-Spearman test for *p* < 0.000) and relaxation instead (rho-Spearman test for *p* = 0.043). Thus, it was confirmed that practical activities during first aid classes that include practicing chest compressions on a medical simulator demonstrate a measurable effect.

## 4. Discussion

Educating children and adolescents in first aid is one of the most important forms of improving health awareness in society today. Knowing how to handle stressful situations significantly increases the chance of responding appropriately and quickly, which is why it is so important to start learning from an early age. This study presents a multidimensional view of teaching CPR to children.

An analysis of the first aspect indicated that there were significant differences in the overall quality of chest compressions among children depending on their age. The data obtained in the study support this claim because there was a statistical correlation observed between the quality of resuscitation and the age of the children. It was demonstrated that 10-year-olds achieved only 24.13% in the overall quality of the compressions, while 14-year-olds achieved more than double of that at 67.61%. Similar results were reported in the studies by Abelairas-Gómez et al. and Ecker et al., who also noted that the quality of resuscitation increases with the age of the child [23,24]. This increase may result from the child’s body mass gain, making it easier to achieve the correct depth.

Another parameter evaluated was the compression depth. It was confirmed that the studied children achieved an insufficient chest compression depth. Matching results were also reported by Jones et al., as well as by Berthelot et al., who noted the failure of children aged 10–12 to achieve an adequate compression depth [25,26]. However, some scientific studies show that children over the age of 10 are able to reach a dedicated depth during phantom exercises [27]. In contrast, a study by Jones et al. also demonstrated that none of the children aged 10, and only one in five children aged 11–12, reached the optimal depth during a simulation on an adult torso [28]. Also, a study by Berthelot et al. confirmed that children as young as 10–12 years old have difficulty reaching the lower depth limit [26].

The results clearly indicate a better quality of resuscitation performed by boys than girls. Female students achieved a significantly lower mean quality of chest compressions (44.69%) than boys in the same age group, who averaged 60.23% in the study. This result was also confirmed by Abelairas-Gómez et al.’s study, showing that young men achieve better chest compression efficiency than girls [23]. Nevertheless, attention should be paid to the specific parameters achieved by students of a particular sex. The chest compression rate was independent of sex (*p* = 0.378); therefore, both girls and boys achieved similar values for this parameter. On the other hand, in the case of evaluating thoracic relaxation, a significantly better result was achieved by girls compared to boys (66.14% vs. 56.78%; *p* = 0.011). This is another argument proving the effects of body mass and strength alone on the effectiveness of the achieved chest compression depth. Considering the foregoing data, it should be considered that each sex should work on a specific criterion of resuscitation (girls should work on depth, while boys should work on relaxation). This can provide a valuable cue for teachers and BLS instructors in schools. A systematic review by Finke SR et al. in 2018 highlighted that the gender aspect of resuscitation training at school age is underestimated. It was shown that girls are significantly more motivated to participate in CPR training (*p* < 0.001), respond to cardiac arrest (*p* < 0.01), have better CPR questionnaire scores (*p* < 0.025) and remember emergency numbers better (*p* < 0.05) [28]. However, these aspects were not the subject of this study.

It was demonstrated that children who claimed to have participated in first aid classes earlier achieved higher scores in overall CPR quality. In a post hoc analysis, the study was able to confirm that experience in conducting chest compressions allows for better compression depth (*p* = 0.040) but has no effect on rate (*p* = 0.464) and relaxation (*p* = 0.428). Thus, it is reasonable to believe that BLS classes allow for long-term improvement in the practical performance of compressions to an appropriate depth, but the ability to maintain pace and relaxation requires more frequent repetition.

In summary, none of the age groups studied achieved the minimum mean depth of chest compressions during adult CPR under simulated conditions. Only single subjects aged 12 and older showed the ability to compress the simulator’s chest to a minimum depth of 50 mm. According to the authors, the results encourage a discussion about the formula for teaching BLS at school. Since children between the ages of 10 and 14 do not show a predisposition to achieve effective chest compressions yet, it makes sense to focus on quickly calling for help from an adult, who will be able to perform qualitatively correct CPR.

Regarding future research or perspectives, the authors recommend developing an algorithm dedicated to children as well as to people who are physically unable to perform proper CPR. The conclusions of this study will be presented to the ERC committee. Feedback provided to children during exercises is also important. Teachers and instructors should make students aware that their compressions are not as effective as those performed by an adult. The use of digital measurements can help customize exercise for a person. The instructor can then point out specific factors that the student should work on to improve the quality of CPR. Future multicenter studies should be conducted and include the analysis of additional variables such as BMI, physical fitness, self-esteem and student satisfaction level.

### The Limitations of This Study

The authors note excluding the group of children aged 15 from the study due to underrepresentation. It is possible that these students would have reached the minimum mean compression depth values. The survey did not include information on time since the student’s last first aid training. This parameter could prove helpful in assessing the short- and long-term effectiveness of BLS classes in schools. The group of students in grades 1–3 of elementary school was also omitted due to the absolute lack of predisposition to perform effective CPR in this age group, as confirmed in the literature. The survey questionnaire omitted aspects such as the child’s physical condition, anthropometric factors (e.g., BMI), diseases, and disabilities [29]. In subsequent studies, it is necessary to consider assessing the correlation of these variables with the quality of resuscitation.

## 5. Conclusions

Age, sex and body mass play important roles in the quality of resuscitation provided by children. In the 10–14 age range, boys achieve a better depth of chest compressions compared to girls, while girls achieve better relaxation. None of the age groups studied achieved the minimum mean depth during adult thoracic compressions under simulated conditions. First aid exercises increase the effectiveness of performed CPR through higher overall quality and superior compression depth, yet the rate and relaxation are not subject to improvement in long-term evaluation. We recommend that, when implementing BLS classes at school, teachers adjust their assessments and requirements based on the psychophysical abilities of children.

## Figures and Tables

**Table 1:** Characteristics of study subgroups.

Age of Participants	Number of Participants [*n*]	Gender [*n*], *p*-Value * (W—Woman; M—Man)	Average Weight [kg]	Previously Completed First Aid Classes [*n*], *p*-Value *	Previously Performed Chest Compression Exercises [*n*], *p*-Value *
10 years	53	W = 33; M = 20; *p* = 0.010	40.32 (SD ± 11.99)	25 (47.17%); *p* = 0.560	19 (35.85%); *p* = 0.004
11 years	62	W = 34; M = 28; *p* = 0.281	43.76 (SD ± 11.37)	42 (67.74%); *p* < 0.000	34 (54.84%); *p* = 0.281
12 years	51	W = 26; M = 25; *p* = 0.843	52.27 (SD ± 15.82)	18 (35.29%); *p* = 0.003	20 (39.22%); *p* = 0.029
13 years	63	W = 34; M = 29; *p* = 0.373	57.14 (SD ± 14.09)	36 (57.14%); *p* = 0.109	36 (57.14%); *p* = 0.109
14 years	46	W = 20; M = 26; *p* = 0.211	61.20 (SD ± 12.43)	43 (93.48%); *p* < 0.000	39 (84.78%); *p* < 0.000

* Chi^2^ test.

**Table 2:** Quality of chest compressions by age group.

Age of Participants	Overall Quality of Compressions [%]	Pace of Compressions		Depth of Compressions		Correct Relaxation [%]
		Correct [%]	[/min]	Correct [%]	[mm]	
10 years	24.13 (SD ± 28.58)	57.72 (SD ± 26.46)	112.81 (SD ± 16.68)	8.23 (SD ± 20.08)	33.96 (SD ± 8.25)	72.64 (SD ± 21.86)
11 years	44.40 (SD ± 30.78)	61.27 (SD ± 20.69)	113.47 (SD ± 7.67)	19.47 (SD ± 25.75)	40.18 (SD ± 8.04)	70.77 (SD ± 27.84)
12 years	58.96 (SD ± 30.85)	62.92 (SD ± 19.82)	114.47 (SD ± 6.89)	32.53 (SD ± 31.22)	44.00 (SD ± 7.95)	55.18 (SD ± 31.40)
13 years	65.54 (SD ± 27.20)	66.37 (SD ± 20.47)	112.17 (SD ± 8.45)	35.81 (SD ± 29.69)	45.97 (SD ± 6.82)	59.46 (SD ± 30.50)
14 years	67.61 (SD ± 27.27)	63.89 (SD ± 24.86)	110.52 (SD ± 10.14)	49.24 (SD ± 33.27)	48.22 (SD ± 7.50)	47.67 (SD ± 33.03)

## Data Availability

The data presented in this study are available upon request from the corresponding author for confidentiality reasons.
